# Transactions of the Imperial Medical Association of Vienna

**Published:** 1845-04

**Authors:** 


### Art. III.

*Verhandlungen der K.K. Gesellschaft der Aerzte zu Wien.—Wien,* 1842-3. *Transactions of the Imperial Medical Association of Vienna.—Vienna,* 1842-3. Vols. I and II. 8vo, pp. 509, 322.

The first of these volumes contains a selection of the papers read to the Society from its institution in 1838 to the close of the year 1841.

A short preliminary sketch of the rise and progress of the association is given at the commencement of the volume by Dr. Knolz. From this, we learn, that it originated, as many similar institutions have done, on occasion of the Austrian capital being threatened by the ravages of the cholera, but that it was not finally organized till the year 1838. The association was then divided into four different sections, of which each member might select one, or more, as his peculiar province:

I. Chemistry, Natural Philosophy, and Natural History.

II. Anatomy, Physiology, Psychology, and Comparative Anatomy.

III. Theoretical and Practical Medicine, Legal Medicine, Pathological Anatomy, and the Veterinary art.

IV. Theoretical and Practical Surgery, Ophthalmology, and Midwifery.

Upon all these subjects, various essays have been brought before the society, and we are here presented with a selection of those which have been considered most worthy of publication.

We observe in the short programme given of the order of business at each meeting of the society, that one point is especially attended to, which might be imitated, we think with profit, in the medical associations of this country. It is enacted that, in the second meeting of every month, a full history of the weather and of the prevailing diseases (Witterung's and Krankheit's Constitution) of the preceding month, shall be presented to the association by the secretary, and the aid of all the members is requested towards the completion of this desirable object.

Ninety pages are next occupied by a condensed history of the meetings of the society during the first session of 1838-9. From this brief notice we learn, that almost every important discovery of the year was brought under the notice of the members, while many points of investigation were suggested, which we subsequently find to have been followed out more completely, and to which perhaps we owe some of the original essays in this volume.

### SECT. I. PHYSIOLOGY.

In this department we find little that is novel; indeed we are surprised to meet with only one paper, on a subject so congenial to the patient spirit of investigation of our German brethren. Nor is the essay of Professor Czermak distinguished by any peculiar originality, and we should pass it over in silence were it not for one or two observations and theories, which if not absolutely new are yet worthy of being put upon record.

*On the rotatory movements in the cells of plants.* This is the subject of Professor Czermak's paper. The fact was first observed, we believe, by Costi in 1772, and has since been studied more deeply by Meyer and Dutrochet. After describing the well known circulation (?) in the cells of the chara and vallisneria spinalis, Professor Czermak favours us with his own theory as to the cause of these mysterious movements. They are entirely dependent, he says, upon the evaporation of the sap itself, for the motion of the chlorophylle globules is accelerated by heat, and is diminished when the temperature is lowered. The experiments, 15 in number upon which he bases this assertion, are certainly very favorable to this theory, but he allows at the same time that the walls of the cells themselves, must by their contractile power, exercise a considerable influence upon the movements of the globules.

### SECT. II. GENERAL PATHOLOGY.

The essays in this department are more numerous and important. The first three papers are by the same author.

I. *On the influence of theory upon medicine,* by Professor Töltenyi. The author first discusses the effect of the study of comparative anatomy and physiology upon medicine. He bewails, as all must ever do, the confusion and the opposing theories which prevail in the medical schools, and gives a lively and amusing sketch of the opinions which at present agitate the pathological world. He combats with spirit and wit the theory of Carus, Oken, and others, that the diseases of mankind are merely a revival or regeneration of the dormant forms of animal and lower life in our organization. That the fetus is to be considered as little if anything more than a mere animal he denies, and seems deeply shocked at the idea, that we should thus be compelled to ascend gradually in the scale of organization, till we become perfect beings. "Were this the case," he says, "the form first assumed should be that of the lowest degree of animal life, and we ought necessarily to pass through *all* the numerous higher stages of organized beings, before we arrive at the perfect man. Thousands and thousands of animal forms thus would come to be the receptacle of the future reasoning creature." We acknowledge that this picture of Professor Töltenyi's is somewhat overdrawn; he has, in his zeal to stem the tide of what he deems dangerous innovation, drawn largely upon the stores of his own creative mind. That great faults and mistakes have been committed by those who have advocated these theories, we readily admit, but that all, including Meckel's beautiful doctrine of *obstructed organization*, are erroneous and futile, we must utterly deny.

II. From comparative physiology our author next leads us to the consideration of comparative pathology. According to the theorists whom he combats, the former study having laid bare, in a manner, the secrets of creation, we are now called upon to admit that the state and condition which we are accustomed to term disease is in fact neither more nor less than a partial development of the animal life, which exists, though latent, in the human organization, at the expense of the higher development which we term *man*. If, then, we believe disease to be merely life existing within life—if it is merely the lower organization predominating and increasing, at the expense of the more perfect development, it follows necessarily that man can never be *completely* the subject of disease till the moment of death; and that, till that fatal hour, the higher life within him is healthy, and the lower alone diseased. How this doctrine holds in reference to fevers, cachexias, &c. is not sought to be explained.

The contradictions and ridiculous assertions of this German school of ideal pathologists (if we may adopt that expressive term), are admirably described; and with a short extract regarding these we will close our notice of this essay:

"While on the one hand disease is compared to animal life in the lowest scale of existence, on the other, certain writers have associated it with the organization of the highest animals, which, in many cases, exceeds that of man himself. Thus, inflammatory fevers have been compared to the natural condition of the carnivora. Thus, because adhesions normally exist between the pericardium and the heart in fishes and in the amphibia, such adhesions, when they occur in man as the result of disease, are forthwith referred to the condition of the above-mentioned lower animals, and hence arises the curious contradiction, that inflammatory fever belongs to the normal state of the carnivora, whilst its product, the adhesion of the pericardium, sinks us far down in the scale of organization, and levels us with the inhabitants of the waters."

Professor Töltenyi's essay, if somewhat partial in its views, may at least be useful in curbing the exuberant imagination of our German brethren. In England we seldom allow our cooler fancies to carry us so far; but, from the increased intercourse with the continent, we cannot doubt that many strange theories will from time to time be transplanted to our soil.

III. *On the influence of pathological anatomy on medicine*, by Professor Töltenyi. While the Professor allows the great benefit we have derived from the researches of Meckel, Baillie, and the French pathologists, he deprecates the zeal of those enthusiasts who, forgetting the careful observation of disease during life, seek only for its manifestations in the body after death, and thus labour to place the study of pathological anatomy on too elevated a position, and to render it the sole aim and end of our endeavours. He laments, too, and we think with justice, the incomplete arrangement of our pathological museums, where often short and imperfect histories of the previous malady are attached to the most remarkable specimens; and observes that preparations of a certain kind are all indiscriminately crowded together, without reference to the diseases which produced them. We acknowledge that this latter mode of arrangement would be somewhat difficult in the execution; but an attempt at it would serve to draw away the attention of the student from the wonders and curiosities of pathology, which but too often are the sole object of his studies, to the morbid processes which produced them; and would remind him that it is the history of disease in the living body, and not the mere alterations it has left behind it in death, which must form the basis of his professional skill. We believe, however, that these remarks will apply more to Germany and France than to our own country.

IV. *Dr. Gruby's microscopico-pathological investigations* have already appeared in several of the journals of this country. As the present essay seems to have been written previous to the publication of his Latin work, we shall not weary the attention of our readers with a repetition of what has been already before them; and we do this with the less hesitation, as the paper before us contains nothing which is not to be found in the larger publication.

V. *On the matter of contagion*, by Professor Berres. Contagion, according to Prof. Berres, may consist of either dry or moist matter: the former may be in the shape of scales, scurf, or abrasions of the epidermis; and the latter a fluid, contained in vesicles or pustules; and, finally, contagion may be propagated by any secretion or excretion from the surface of the body.

The essence of *dry* contagion exists in an aggregation of semitransparent grayish white globules, about $\frac{1}{10000}$ of a Vienna inch in diameter. These globules swell somewhat in water, but exhibit no further internal structure. In the moist form of contagious matter, we observe a vesicle filled with a clear fluid, which exhibits no traces of organization; but should it become in the slightest degree discoloured, then a number of grayish white, round molecules appear in the fluid. These are about $\frac{2}{10000}$ of an inch in diameter, and contain a small cavity, apparently filled with a delicate vapour. But should the contents of the vesicle or pustule become more turbid,then the large spherical pus-globules become visible.

It is well known that certain fluids are at one time contagious and at another totally inefficient; and the microscope has hitherto signally failed in discovering the cause of this remarkable variety of effect. From the result of his observations, Professor Berres contends that all fixed contagions are, at their origin, alike in form, and that they consist of larger or smaller globules, which, in the moist variety, are surrounded by a clear fluid; moreover, that we have no data to explain the extreme variety in the effects and operations of contagion; and, lastly, that we must allow of a specific life and separate existence to *contagion*, which combines itself with the globules above referred to, and employs them as the means of transport of its hidden power.

Professor Berres conceives contagion to be propagated in two different ways, the material and the dynamic or vital. In considering the first-named mode, our attention should be directed to the anatomical constituents of contagion, as before described, and also to the surface of the human body, as its destined recipient. Professor Berres denies, or at least strongly doubts, the possibility of dry contagious matter acting through the epidermis; and even in the moist form, it would require a large quantity for the necessary endosmose:

"We must, then, allow," he says, "that it is not the materies of contagion, but its specific life or vitality, which constitutes disease; and infection is then a dynamico-vital process, which takes place between living contagious matter, and the individuals predisposed to receive it. If this be true (?), it follows that contagion in all respects resembles the process of fecundation; where it is not the semen itself, but the aura seminalis, which operates on the germinal vesicle."

Continuing this train of reasoning, the Professor promulgates a still more curious theory, viz. that the reciprocal operations of the contagion, and of the recipient, closely resemble the office of the semen of the male and of the germinal vesicle of the female in the process of generation.

After giving at considerable length a description of the minute structure of the skin, Professor Berres proceeds to discuss the validity of Bulard's assertion, that the oriental plague is a disease arising from absorption of the poison by the lymphatics. This Professor Berres denies, and he doubts whether, in the majority of cases, either the veins or the lymphatics, are the bearers of the contagion, but he again asserts that in the plague, as in most other diseases, infection is to be considered as a dynamico-vital process, similar to the supposed one of the aura seminalis.

We cannot venture to assert that the difficult question of contagion has received much illustration from the essay of Professor Berres. His histological descriptions are indeed excellent; but, like the rest of his countrymen, he is apt to wander beyond them into a maze of theory, which greatly detracts from the acknowledged value of his microscopical observations.

VI. *On pathological chemistry*, by Dr. J. F. Hellers. This exhortation to the study of pathological chemistry contains an exposition of the author's views upon the causes of disease: it will interest particularly the rising generation of medical men, at least that portion who regard the human body as one vast chemical laboratory. Dr. Hellers traces, in a brief sketch, the rise of chemistry from the alchemist, through the study of the constituents of the mineral and vegetable world, up to its present high development in the study of the chemistry of the human body (anthropochemie), which he again subdivides into the physiological and pathological branches.

Dr. Hellers then gives his own opinions regarding the origin of disease, which, however, he very candidly assures us, are only to be regarded in the light of an attempt at laying down some fixed principles of pathological chemistry, and that his assertions and theories here expressed are only an approach to, or an attempt to arrive at, the truth.

Disease may arise in the human body:

1. From too high a state of oxidation of certain substances during respiration and circulation.

2. From the excessive diminution of the supply of oxygen, as is shown by the state of the blood in scurvy, chlorosis, cholera, &c.

3. By preponderance of the electro-positive or of the electro-negative condition; the former causing to predominate the *alkaline*, the latter the acid character in disease, as we see exemplified in the varying conditions of the urine and other secretions.

4. By abnormal aggregation of certain portions of our organism, as by an increased secretion of fluid in dropsy, diabetes, &c., or by a diminution, on the other hand, of the same fluids, causing deposits in various organs.

Dr. Hellers concludes by earnestly calling upon the profession to aid him in his endeavours to fix the principles of pathological chemistry on a sure basis, which can only be accomplished by the united labour and observation of the whole medical world.

### SECT. III. HISTORY OF EPIDEMICS.

This section comprises the history of disease and the epidemic constitution of the years 1838-39-40. It must be acknowledged that, in the accuracy and perfection of statistical details, we are considerably surpassed by our German brethren. The regular statistical accounts that are published in the continental journals, embracing the whole history of the ravages of disease during the preceding three months, half year, or year, are seldom imitated in England. We believe that the disunited state of the medical profession in this country, perhaps renders it difficult to accumulate the requisite data; but in our dispensaries and larger hospitals there is good opportunity for so doing, were our attention once properly directed to the subject.

I, II. The first two papers of this section relate entirely to the sanitary condition of the Austrian capital, and from the nature of the subject it is not possible to give an analysis of their contents. No epidemics of any serious importance have occurred during the period embraced by the report.

III. *On the means of forwarding the practical study of epidemic disorders*, by Dr. H. H. Beer. The incomplete acquaintance we as yet possess of the true nature of epidemics has given rise to endless theories, and to the most bitterly contested discussions among medical men. To render the history of epidemic disease practically useful for sanitary purposes, Dr. Beer proposes, 1, that all the medical profession should unite to furnish good comparative histories of epidemics; and, 2, that there should be a constant renewal and revision of approved medical topographies, with a full account of the genius *epizooticus*, as well as of the prevalence of blight or other disease among plants, at the same time. By these means, steadily pursued, Dr. Beer thinks that, after years perhaps of patient perseverance, we may become thoroughly acquainted with the history and true nature of epidemic disease.

IV. This is a short notice of Dr. Bulard's attempt to ameliorate our sanitary regulations regarding the plague. Professor Wirer, the author, thinks that three or four days of quarantine are quite sufficient for travellers, but that more perhaps should be required for certain varieties of merchandise. The great prevalence of plague in Turkey he ascribes to deficient drianage of the soil, and to the generally neglected state of agriculture. Until these obstacles are removed, the plague will continue its ravages in these countries.

V. This is upon a similar subject, and from the pen of Dr. Knolz. Dr. Knolz claims, for Austria, the merit of having first established regular quarantine, and also for having been the first to diminish its duration from forty-two to twenty days. The chief object of Dr. Knolz's essay is to examine the propositions of Dr. Bulard, that important alterations should be made in the sanitary laws.

The plague in Egypt is supposed to arise from an accumulation of causes, all tending to the development of the disease. The exhalations from the marshes, the rising temperature of the spring, the imperfect mode of interment of the dead, and the misery and uncleanliness of the natives, all concur, according to Malfalti, to induce the most fatal epidemics among cattle, which are almost always observed to precede the outbreak of the plague among the inhabitants of the Delta. It is uncertain whether these malignant disorders are transmitted from the lower animals to man, or whether the plague is to be traced as originating in the Dan el Maja, of the Delta, which is a most malignant form of intermittent fever, often proving fatal at once, or in a second paroxysm with delirium and intense headache. Petechial typhus soon makes its appearance and is accompanied by carbuncles and the buboes of the plague; and thus the author believes the transition is probably accomplished, from simple marsh fever to the most complete oriental plague. It is then exceedingly difficult to distinguish endemic cases of plague from those propagated by contagion; and indeed as soon as the real cases of plague make their appearance, each house wherein they occur becomes a fresh focus of infection. Dr. Knolz considers Dr. Bulard to be in error in the following particulars: 1, that he denies the plague to be a purely contagious disease; 2, that he considers all the hitherto-adopted modes of treatment as totally insufficient; and, 3, that he denies altogether the great use of a free current of atmospheric air as a disinfecting agent. But as we have already stated our views at length on the whole question of the plague and quarantine, we need not accompany our author further in his criticism.

VI. *On the epidemic of abdominal typhus in Vienna, during the months of July and August* 1838, by Dr. Dobler. This epidemic was mild in character, and strictly confined to two districts in the suburbs of the Austrian capital. It does not appear to have been contagious. The majority of those affected were from twelve to thirty-five years of age; but neither children nor the aged entirely escaped. Violent cases with head symptoms, were rare; but some patients sank rapidly after five or six days' indisposition. The symptoms, as detailed, were those of the typhoid fever of Paris. In some instances, the strongly-marked evening exacerbations and remissions, exhibited completely the type of intermittent fever. Two individuals lost upwards of seven pounds of blood by hemorrhage from the bowels; but they both recovered, after a tedious convalescence. Relapses were very frequent, and greatly increased the danger. The mortality was small, as compared to the number of cases, nor were the sequelæ of the malady of great importance.

Dr. Dobler ascribes the epidemic in a great measure to the high temperature of the spring of 1838; and also chiefly to the exhalations from a sewer or small brook which traverses the suburb of St. Ulrich and Spittlberg, to which municipal districts the fever was confined. No beneficial results were obtained by antiphlogistic treatment, and bleeding was especially injurious. The only remedy that proved really efficacious—and *this in all cases*—was crude alum, given in the form of powder, and in doses of from two to five grains every hour. Under every train of symptoms—diarrhea, delirium, debility—the alum proved equally beneficial.

VII. *Remarks on the epidemic appearances in the summer of* 1838, by Dr. C. Sterz. Dr. Sterz fully corroborates the statement of Dr. Dobler as to the beneficial effects of crude alum in the abdominal form of typhus fever. It was especially valuable in checking the exhausting diarrhea, and apparently acted as an astringent and tonic upon the relaxed and ulcerated mucous follicles of the intestines. The muriate of iron, which by some has been vaunted as a specific in typhoid fever, was, in Dr. Sterz's hands, inert, exerting no influence on the secretions of the bowels, save that of blackening the fæces.

As a remarkable instance of severe secondary disorder after fever, Dr. Sterz relates the history of a case of noma, accompanied by extensive necrosis of the bones of the face. The child, a boy of 10 years old, recovered at last, but with the loss of almost the entire superior maxilla on the left side, leaving a hideous gap, only partially covered by the cicatrix of the ulcerated cheek.

VIII. *On the epidemic of abdominal typhus, as observed in the wards of the General Hospital of Vienna, in* 1838, by Dr. C. Folwarczny. This consists chiefly of a statistical account of the cases; from which we find that, out of 115 patients—55 of whom were males and 60 females—there occurred 30 deaths; 13 males, 17 females. The treatment, in all severe cases, and whenever there was diarrhea, was solely by crude alum, but the milder forms yielded to an infusion of ipecacuanha and acid drinks. Typhoid ulcerations, so admirably described by Rokitanski, were found invariably in the ileum.

IX. *Observations on the epidemic fever of the year* 1839, by Dr. Wirer. This appears scarcely to have deserved the name of an epidemic, and to have been remarkable only from the frequent occurrence of miliary eruption on the body during its course. The treatment adopted was chiefly emollient, with cooling lotions to allay the heat of the skin. Alum is not mentioned as being employed.

### SECT. IV. SPECIAL PATHOLOGY AND PATHOLOGICAL ANATOMY.

I. *On the bite of venomous serpents*, by Professor J. J. Czermak. Dr. Czermak's experiments were made with vipers (vipera rhedi,) from Dalmatia. He found that the amphibia, probably from their slow and less perfect circulation, resisted the action of the poison longer than the mammalia, and much longer than birds. The poison he believes to act entirely through the blood, disorganizing that fluid, and rendering it incapable of coagulation.

II. *On endocarditis, with reference to a case of inflammation of the semilunar valves of the pulmonary artery*. The foramen ovale was also open in this case; and the lesion of the valves of the pulmonary arteries was greater than in any instance we have heard of.

III. *A case of complete ossification of the semilunar valves of the aorta*. This does not contain anything of peculiar interest.

IV. *On the eye as regards diagnosis*, by Dr. J. F Frank. Few will deny that the dependence of the ancient physicians on the signs afforded by this organ in disease were more or less founded on careful observation. Dr. Frank, from the results of forty years' experience, fully corroborates this Hippocratean doctrine, which has no doubt fallen into neglect in our days, from other and more certain signs of disease having become known. The mere dryness, filminess, or bright glassy appearance of the cornea and sclerotic may, we think, often result from very opposite conditions of the body, and cannot indicate with certainty any peculiar disease. In connexion with the general aspect of the countenance, we readily allow to the eye all its influence, but singly it is a sign of little value.

### SECT. V. PHARMACOLOGY AND THERAPEUTICS.

I. *On electricity as a remedial agent*, by Professor Wisgrill. After referring to the great vogue electricity enjoyed in medicine at the beginning of the present century, and to the subsequent contempt into which this remedy fell, Dr. W. asserts that its neglect arose, less from the inefficacy of the means, than from the mode in which they were employed. A revolution in favour of this powerful agent has now, he says, taken place, and this has especially been aided by the discovery of magneto-electricity. The employment of electricity in medicine has not hitherto, according to Dr. Wisgrill, been based upon scientific principles. By the profession in general, the study of natural philosophy has been regarded merely as preparatory, and has not been directed with reference to the practical part of medicine. Moreover the apparatus hitherto used has been costly and difficult of application, while, at the same time, it was essentially incomplete. We may observe, however, that the rotatory apparatus of *Kail*, which was used by Dr. Wisgrill, is inferior to that now generally employed in England. During the years 1837 and 1838, Dr. Wisgrill had treated twenty-one cases by magneto-electricity. We cannot attempt more here than to give the results obtained. In four cases of *hemiplegia* (two of which were complete,) great and decided benefit was obtained, by daily applications of the machine. In one case of *hysteric clavus*, the evil was arrested for ten months, and then having returned for twenty-four hours, was again finally put a stop to without difficulty. The remedy is of little or no avail in *toothache*, as we ourselves can testify ; but in four cases of *infra-orbital neuralgia* its effects were truly surprising. One of these cases had continued for twenty-five years, and latterly without intermission. During five months the application of magneto-electricity was persevered in, at first once a day, and for a short period and subsequently twice a day, for a full hour each time. The result was, that the pain ceased almost entirely for six weeks, and during the succeeding five months it recurred but four times for the space of ten days on each occasion, and generally in consequence, as Professor Wisgrill believes, of atmospheric changes.

II. This is an essay on the same subject, by Professor Wirer. The author speaks still more confidently than his colleague, as to the beneficial effects of magneto-electricity in paralysis of long standing. Professor Wirer further states, that in all cases where a beneficial effect has been produced by this remedy, a degree of febrile action is set up in the system, which he compares to that observed when salivation ensues from the use of mercury or of the preparations of gold. In the cases of paralysis to which he refers, this peculiar febrile action always preceded the beneficial operation of the remedy.

III. *On the employment of fat as a therapeutic agent*, by Professor Wirer. This article is merely a strong recommendation to its further use in disease, and especially in those complaints in which it has been successfully employed. The author refers to the investigations of Liebig and Rettenbacher regarding the physiological use of fat in the human body, and concludes by expressing his earnest desire that more attention may be paid to this department of therapeutics. "Has not," he asks, "every species of fat its own peculiar properties and action on the human body? and is it not desirable, that many now obsolete remedies, consisting entirely of various animal fats, should be again received into the 'materia medica?'"

### SECT. VI. MIDWIFERY.

I. *History of a case of triplets*, by Dr. Bartsch. Both the mother and children did well, though placed in great danger, from threatened convulsions preceding the birth of the first child, from the transverse position of the second, and from hemorrhage during the birth of the third infant. The woman was 38 years of age, and had previously borne nine children. She had twins three years before the triplets appeared.

II. *On the influence of the imagination of the mother upon the fetus in utero*, by Dr. Ernest Freiherr v. Feuchtersleben. Dr. von F. states that the existence of such an influence was never questioned until the commencement of the eighteenth century, but that when once doubted of, it was speedily denied, and that which before was regarded as an undoubted fact, now became a fable unworthy of our science. But within the last twenty or thirty years the spirit of calm philosophical inquiry has forced us to admit that many instances strongly in favour of the existence of such an influence are now upon record, and the question to be solved is rather, how could such things be produced if such influence be denied? The opponents of the theory believe that they may occur from long continued pressure upon the uterus during pregnancy; from an injurious position of the fetus in utero, arresting the normal development of this or that portion of the body; from disease of the fetus itself; from an amalgamation of one or more of the parts of one or more fetuses with another, &c. The author, without avowing his own creed upon the subject, believes that this question can never be definitely settled without a more perfect knowledge of those causes which influence the arrest of development in the fetus, and especially as to the period, if any, in fetal life, at which this influence ceases to exist.

### SECT. VII. SURGERY.

I. *Results of lithotrity, and of attempts to dissolve urinary calculi in the bladder*, by Dr. Graf. Dr. Graf has only employed lithotrity five times, in four of which he was successful. Dr. Graf announces that he has discovered a means of dissolving urinary calculi in the bladder. Dr. Graf states that he became acquainted with this remedy by chance, and that sooner or later he will make it public. It was peculiarly powerful upon phosphatic calculi. Is not all this very redolent of quackery? How came the Society to tolerate it?

II. *Report of the Orthopedic Institution at Vienna*. By Dr. August Zink. In every large town such establishments are now being formed:

that of Vienna was founded in 1838, and the present report embraces the first two years of its existence. The majority of the patients were females, and 56 out of 119 were affected with contorted spine; 14 suffered from contraction or deformity of one or more limbs; 16 exhibited a decided tendency to chlorosis or to scrofula; 5 had hemiplegia, or general muscular debility; and 28 entered the Institution to invigorate their weakly constitutions, or for the purpose of increasing their stunted growth. The proportion of females affected with spinal contortion was, to that of males, as 22 to 1. Dr. Zink appears to have bestowed much attention to that one form of spinal contortion which has been termed the sigmoid flexure of the column. It generally commences from the 5th to the 8th year of childhood, between the third and fifth dorsal vertebræ, and the deviation is almost invariably towards the right shoulder-blade. On careful examination at this period we discover that the right shoulder exhibits an excess of nutrition over the left, and this is apparent even in the bones, and especially in the right shoulder-blade. In consequence of this, such children exhibit a remarkable tendency, in spite of all admonitions to the contrary, to lean towards the weaker side, and this even during sleep, while on every occasion requiring muscular exertion they prefer the stronger hand.

"From repeated observations," says Dr. Zink, "I am convinced that the greater part of the mischief here detailed is effected during sleep, and consequently is in operation during one third of the patient's daily existence. The head then leans down towards the left side, and the lung on that side is compressed, one lung only (the right,) performs its full office, and the muscles of respiration on that side are in a state of activity greatly exceeding that of the left. .... I have also ascertained that the convexity of the deviation of the spine accords exactly with the insertion of those muscles which are most active in the process of respiration. This deviation from the perpendicular, so high up in the spinal column, is often overlooked, and the inferior and secondary contortion to the left side in the lumbar vertebræ is often regarded as the primary affection."

This form of scoliosis occurs chiefly among the children of the wealthy, and is much more frequent among females than males. It is curable as long as the vertebræ can be brought back to their natural position. Dr. Zink lays much stress upon the necessity of attending to the position of the body during sleep, and he believes that many attempts to cure are unsuccessful from the injurious posture being resumed during the period of repose. The watching of the patients during sleep, as may well be imagined, has been the most tedious and costly portion of the cure. During the day the ordinary gymnastic exercises were employed, taking care that the weaker side especially should be kept in constant action. Dr. Zink believes that he has obtained much benefit also from frequent singing, which dilates the whole chest equally. Many girls entered the Institution on account of chlorosis, or a tendency to that disease. It was remarked, that if the menstrual secretion had already commenced in these patients, it was almost always suppressed during the period of their stay, and this without the slightest drawback to their daily improving health.

III. *On tenotomy as an orthopedic means*, by Dr. Zink. In this brief notice Dr. Zink praises highly the subcutaneous method, and states that he has in several cases, and with success divided the muscles of the spine from their attachments, and thereby effected the cure of several almost hopeless cases of scoliosis.

IV. *Experiments with vaccine lymph, obtained from Dr. Reiter of Munich*, by August Zöhrer, surgeon to the Imperial Foundling Hospital in Vienna. The only point worthy of notice here is the observation of Dr. Zöhrer, that the most certain and complete indication of true lymph having been employed is, that the vaccine vesicle carries off in its scab, both the dermis and the bulb of the hair, and leaves the cicatrix of a reticular form, with many deeper small points or holes. The lymph here employed had been obtained from the cow by Dr. Reiter of Munich, and had been once transmitted through the human body before it was used by Dr. Zöhrer.

SECTION VIII. ON MINERAL WATERS.

I. *On the employment of the Carlsbad waters*, by Dr. Sterz. Dr. S. warns his brethren against the indiscriminate use of these waters, and especially of the Sprudel spring, which he asserts usually tends to increase abdominal congestion, instead of dissipating it, as generally believed. It has long been known that the waters of Carlsbad are contraindicated in all acute diseases, and Dr. Sterz now denies the use of the Sprudel spring to cases of abdominal and venous congestion. He concludes his short essay with the history of two cases which certainly would go far to establish his opinion in this respect.

II. *On the good effects of the Carlsbad waters in cases of diabetes mellitus and insipidus*, by Dr. Hochberger.

III. *Elephantiasis cured by the Carlsbad waters.* These are certainly to all appearance well authenticated instances of recovery from the disorders named under the use of the Carlsbad springs ; but the history does not present any points of peculiar interest.

IV. *On the good effects in disease of the whey, the water, and of the atmosphere of Ischil*, by Dr. von Wirer. This is certainly somewhat of a quaint title, but Dr. von Wirer is honest enough to allow that his patients, most of them the victims of the dissipation of the Austrian capital, during the preceding winter, are as much benefited by pure air and exercise, as by the whey which they swallow in such considerable quantities.

The first fifty pages of the second volume contain an abstract of the Society's proceedings during the session of 1841-42, the fourth year of its existence. It is evident that the increasing years of the Institution have not chilled the fire of scientific investigation, and that delightful harmony, so conspicuous in the reunions of our German brethren, appears to have been uninterrupted.

I. *On the value of systems in medicine*, by Dr. Knolz. After a long preamble on the tendencies of medical science during the last two thousand years, Dr. Knolz discloses to us that in the eighteenth century the doctrines of Hippocrates were on the point of becoming permanently triumphant, when their victorious course was arrested by the schools of Brown and of Hahnemann.

"The Scottish physician denied to pathology the participation of the humours of the body in disease, and he contracted within the narrow limits of asthenia and hypersthenia all that host of maladies which had been so learnedly arranged and described by Sauvages, while even his simplifications of our own science were in their turn overthrown by Hahnemann. True inflammatory disorders were to these men nosological rarities; they denied the existence of hereditary disease; they passed over in silence the numerous host of local disorders; and, finally, repudiated the noble Hippocratean doctrine of crises in disease, and of the vis medicatrix naturæ. While the Scottish innovator confined his practice to wine and opium, the German Homœopathist swept away at once the sciences auxiliary to medicine, and reduced the practitioner to the bare observation and arrangement of symptoms, while at the same time he extended the operation of drugs far beyond the bounds of possibility."

Dr. Knolz gives a fearful picture of the devastations caused by Brown's doctrines on the continent of Europe, and especially in Italy, while he acknowledges that in England they found small favour from the beginning. But the doctrines of Hahnemann cannot in this respect be compared to those of Brown, for the former have never gained ground in the schools, nor have they been adopted by a single eminent man. The system of Brown, according to Dr. Knolz, would not have declined so rapidly, had not a more worthy opponent entered the field; and the doctrines of asthenia vanished before the study of natural philosophy and of its various branches. The author concludes by earnestly recommending the Hippocratean doctrines of experience and science combined, as the only sure basis of study; and reminds us that if in ancient times medicine was a *divine art*, it is in modern days the highest of the sciences.

II. *On the object and the organization of medical societies*, by Dr. Sporer. This is a mere exhortation to form medical societies for the investigation of disease. Rules are laid down for their due government and efficiency, which could scarcely be adopted here.

### SECT. II. HISTORY AND GEOGRAPHY OF DISEASE.

It is in statistical information, of the kind contained in this section, that our continental brethren so much excel us in England.

I. *On the characters of disease in Vienna during the year* 1841, by Dr. F. v. Feuchtersleben. Though extremely useful in his own locality, still this essay does not present any points to interest the British reader. There are, moreover, in the paper various expressions which puzzle us not a little, such, for instance, as the term "*lateral character of disease.*" Vienna does not appear during this period to have been visited by any epidemic, and the usual disorders of this great capital exhibited no tendency to increased virulence. Of inflammatory disorders pneumonia appears to have been exceedingly frequent.

II. *On cretinism*, by Dr. Knolz. This is a valuable paper, by one who has enjoyed every opportunity of studying this difficult disease. Dr. Knolz briefly reviews the opinions of previous writers, and observes that they all carefully avoid answering, in a direct manner, the question that at once suggests itself, *what is cretinism?* The definition of the malady, as given by Akerman, by the Wenzels, and by Foderée, and others, he regards as erroneous. The characteristics they describe are by no means constant. Thus they speak of the complexion as being always of a leaden hue, and the thyroid gland as being always very much enlarged; while, in very many instances, perfect cretins are seen with the usual bronzed healthy look of the mountaineer, and whose thyroid gland does not in the least exceed its normal dimensions. Dr. Knolz has been enabled, by a ten years' residence among the cretins around Salzburg, in the Tyrol, to give the following description of the malady.

The entire body of the cretin bears the marks of insufficient or irregular development. The average stature is far below that of the Caucasian race; the head is small in proportion to the size of the body, and, from its peculiar form, resembles rather that of the lower animals than of the perfect human being; as the lower portion of the face, containing the organs for the reception of food, are infinitely more developed than those allotted to the senses. The facial angle usually approaches more nearly to that of the orang outang than to that of man. The occiput is generally flat, and the back part of the skull runs parallel with the spine, as in ruminating animals. The occipital foramen, probably from deficiency of the cerebellum, lies very far back in the skull; and this, no doubt, is the cause of the head falling so far forward upon the chest. The face is usually broad and short, as are also all those features which give to the countenance its intellectual character. The eyes are sunk in the head, and widely separated; the eyelids heavy and overhanging; and the forehead retreating and low. The jaws are extremely prominent; the mouth wide, with thick sensual-looking lips, from which the saliva constantly flows over the scarcely perceptible chin. Dr. Knolz entirely denies that the dimensions of the thyroid gland afford any just measure of the amount of cretinism in individuals so affected. From the peculiar position of the spine, in relation to the thighs, the cretin's gait resembles not a little that of some of the monkey tribes when walking erect. Ipzhofen denies that the genital organs in cretins are unusually large; but Dr. Knolz confirms, in this respect, the statement of others, and asserts their excessive development to be a universal law.

With respect to the physiological qualities, the digestive organs of cretins greatly preponderate in activity over the others; the whole occupation, the whole life of the cretin is centered in the enjoyment of eating, though Dr. Knolz does not think, with the Wenzels, that their digestive powers are necessarily in proportion to their enormous appetites. The liver in cretins, as in the lowest class of animals, is of great relative size. They undoubtedly possess muscular irritability, and also the faculties of thought, and of will, but all in a very inferior degree. In the complete cretin, the power of speech is almost entirely, or is quite, extinct; nor does this result from deafness, for he is equally unable to learn the finger-language of the deaf and dumb.

The true characteristics of the cretin are then to be sought for in the head, both in the skull and in the face; and to these may be added, the undue development of the liver, and of the sexual organs. Fetal life in the cretin may be said never to be extinguished; he is never developed into the man, scarcely rising above the condition of the merest infant, save by the great activity of the digestive and sexual organs. In fact, they otherwise possess merely a vegetative life; and, were their appetites and power of locomotion taken away, they would be no more, observes Dr. Knolz, than warm-blooded plants. The disease of cretinism Dr. Knolz considers to arise from undue activity of the ganglionic or vegetative, with a deficiency of the cerebral, system; and not, as many have imagined, from any peculiar induration of the whole or of parts of the brain, or from rachitis, syphilis, &c.

On the whole, Dr. Knolz's essay is well deserving of an attentive perusal, and bears the marks of a mind bent upon the investigation of truth.

IV. *On the sanitary statistics of Steyermark* (*Styria*), by Dr. Onderka. We cannot praise this account of the wild mountainous regions south of the Danube: it is written in so fulsome a style, as involuntarily to remind us of the singular histories in the 'Miscellanea Curiosa,' or the still more remarkable works of this kind of the fifteenth and sixteenth centuries. Nor are we better pleased with the plan followed in this paper, where, one disease being named, all those localities are placed after it where it may have occurred during several years. Dr. Onderka should curtail his exuberance of language, and amplify his statistical information, if he would benefit his readers. The number of medical men in proportion to the population is as 1 to 2512, and in Gratz, the capital, as 1 to 616. The number of surgeons exceeds, by three times, that of the physicians; and they, as must ever be the case in thinly-populated districts, exercise in the mountains all the duties of the general practitioner in England.

SECT. III. MATERIA MEDICA.

I. *On the administration of new chemical remedies*, by Dr. Netwald. This is a paper of mere local interest, and, in fact, is a sketch of the condition of the apothecaries in Vienna. It contains little that deserves our notice, save that we find a description of an institution in Vienna which must, we think, be even more efficacious than our Apothecaries' Hall in upholding the purity of drugs. A certain number of the apothecaries of the Austrian capital have associated themselves together, and founded a depôt or warehouse for medicines, and each member is bound to furnish certain chemical preparations and medicines to this central institution. All these, before they are admitted, are carefully tested by a committee of competent judges; and thus each member is certain that he receives, pure and unadulterated, those preparations which he may not have leisure or opportunity to make for himself.

II. *On the advantages of the infusion of arnica in paralysis of the bladder*, by Dr. Meyer. Dr. Meyer regrets that this plant should now be so much neglected in medicine, as to be in some parts of Germany merely a popular remedy. He relates, at very considerable length, a case where its employment was certainly attended with most beneficial results. The paralytic affection of the bladder, which was of two months' duration, disappeared after three days' use of the arnica infusion; and when it recurred, two months after, it was again cured, in the same space of time, and by the same remedy.

III. *On the advantages of steam-baths, and in particular of the "Sophien bad" at Vienna*, by Dr. Sterz, sen. The diseases most benefited by these baths are rheumatic pains without fever, gout, and its consequences, especially herpetic eruptions, and also ancient indurated glandular swellings, which soften and disappear. In diabetes, the determination to the skin occasioned by the steam-baths, was followed by the very best effects. We are not aware that steam-baths, on the large scale, as they are used on the continent, exist in this country. An attempt was made to introduce them into London some years since, by a gentleman from Vienna; but the attempt did not meet with the encouragement it deserved. It is a pity that, in this age and this country of steam, so powerful and so agreeable a means of acting on the system should not be adopted.

IV. In the next paper, on the same subject, Dr. Gotzy warns his professional brethren against the indiscriminate employment of steam-baths, especially in cases where active venous congestion is going on. Dr. Gotzy's essay is a long one, and develops amply the views of the Vienna school upon dyspepsia and congestive plethora, though we cannot say that it discloses any facts, new to the readers of this Journal.

### SECT. IV. SPECIAL PATHOLOGY.

I. *Explanation of a passage in Mercatus's work, ' De Morbo Gallico,'* by Dr. Pasquali. The substance of this communication is, that Mercatus suspects the venereal poison to centre in the *liver*, and Dr. Pasquali relates three or four cases which would seem to him to corroborate this theory of the Portuguese physician! We do not deem it necessary to enter into further details, especially since the cases he relates are, as may be anticipated, by no means confirmatory, in our judgment, of Mercatus's views.

II. *History of a case of hydrophobia*, by Dr. J. Hassinger. The case is chiefly remarkable from the fact of the bite having been received two years before the appearance of the malady, constituting a third case of this kind within the last two or three years. The two others occurred in this country; and in all the cases, little or nothing was discovered on dissection.

III. *History of five individuals poisoned by arsenic, with remarks on the use of that poison by the mountaineers of Austria and Styria*, by Dr. A. Flechner. In April 1838, five individuals, residing close to some mines at Schögmühl, in Lower Austria, were attacked with symptoms of gastritis. The fever was slight, but there was much depression and debility. The author suspected the water of which these individuals drank to be the common cause, as they did not all eat together. He was confirmed in this supposition by the fact, that a large quantity of cobalt, containing arsenic, had lain close to the spring during the winter; and he suggested that a portion of the arsenious acid in the cobalt ore had been dissolved by the snow and rain, and had filtered through the earth into the spring. The hydrated peroxide of iron was judiciously employed as a remedy in all the five cases; and as the evil effects seem to have resulted from repeated small doses of the mineral poison, so in the same way a few grains of the above remedy were given at short intervals. The beneficial effects of the antidote were almost immediately felt, and in a few days the whole five individuals were restored to perfect health. The water of the spring on being tested in the usual way, exhibited abundant traces of arsenic.

Dr. Flechner then proceeds to give an account of the use of arsenic among the Styrian mountaineers. From the time of Mithridates, wonderful histories have been frequently related of individuals who had by degrees habituated themselves to the daily use of the most dangerous poisons. But Dr. Flechner is, we believe, the first who, from his own authority, informs us that arsenic is used as a popular tonic. It is taken by the hardy Tyrolese and Styrian hunters, in doses which would certainly be most injurious to the generality of mankind. Some swallow as much as two grains of the mineral before they set out on their dangerous excursions in the mountains; and all affirm that they acquire thereby a great increase of bodily vigour and a power of enduring fatigue which they could not otherwise obtain. We will own that Dr. Flechner's account of this Mithridatean practice of the Tyrolese is somewhat startling; and though written with apparent good faith, he does not seem to have himself witnessed the actual employment of the poison after this singular fashion. We are most anxious for further details.

IV. *History of two remarkable cases*, by Professor Wagner, of Lemberg. The latter of these is worthy of notice, as being of rare occurrence. The patient, a girl of 8 years old, and born of healthy parents, was observed at birth to have a small tumour on the right side of the nose, near its root. The tumour was then only the size of a hazel-nut, insensible to the touch, and covered with a skin similar to that of the neighbouring parts. Before the child was a year old the growth had increased to the size of a pigeon's egg, and had become dark coloured and tender. Eight years elapsed before medical aid was sought. The child then appeared healthy, and all the functions were perfectly normal. The tumour now extended from the inner canthus of the right eye over the cheek as far as the right ala nasi, and, passing across the mesial line, covered also a portion of the left side of the nose. It was seven inches in circumference at the base, two inches in diameter, and four inches in length; its consistence soft and pasty; and the colour brownish red, with several white cicatrices. The eyelids of the right eye were partially everted, and inflamed; while the bulb itself was thrust outwards towards the external canthus. The patient complained of a dull pain, extending upwards to the brain, when the tumour was much pressed or handled. The plan of treatment pursued was not such as would have been adopted in England. From the great apparent vascularity of the tumour, Dr. Wagner and his colleagues would not venture upon extirpation by the knife; but it was determined to carry shallow incisions over the surface, and then to excite suppuration and the destruction of the whole by strong corrosives. This was done; but the application of the solid nitrate of silver gave rise to severe symptoms of cerebral reaction, and to an erysipelatous inflammation of the cheeks. But the treatment was still persevered in for ten months and more, though, in spite of all their efforts, new growths were constantly put forth to supply the place of those that had been destroyed by the butter of antimony, and other caustic applications. About the beginning of April, ten months after the patient had first entered the hospital, a great increase of the constitutional symptoms took place, and masses resembling cerebral substance came away from the profusely suppurating surface. On the 10th of April the patient was seized with violent general convulsions, which soon ceased; but there remained a complete paralysis of the *left* side of the body. These convulsive attacks frequently returned, until the death of the patient on the 2d of May. The examination was commenced within five hours after dissolution, and the details are given at great length. The right anterior lobe of the brain was found to have constituted the tumour observed externally during life; a prolongation of its substance, to the extent of five inches in length and an inch and a half in diameter, passed out through the right orbital foramen into the face, having destroyed a portion of the frontal, the nasal, and ethmoid bones, to form a canal for its exit. The membranes of the brain exhibited a high state of inflammation.

The remaining five short essays, which terminate the volume, do not contain anything to interest our readers.

On the whole, we congratulate our Viennese brethren on these first-fruits of their labours; and we trust that every succeeding year will increase their numbers and value. We rejoice, too, to find how much the fond tendency to theorize, formerly so conspicuous in Germany, has been superseded by the spirit of patient investigation. It is true that the new sciences, as we may call them, of microscopical anatomy and physiological chemistry, have opened a fresh field for exuberant imaginations; but the sober earnestness of the work before us is a pledge that the Vienna school will go on in the line of patient observation and study; and we may reasonably hope that the ensuing volumes of these Transactions will equal if not exceed in value those of which we have here given an account.

---

